# Boosts for walking: how humorous messages increase brisk walking among cognitively fatigued individuals

**DOI:** 10.1186/s12889-023-17464-z

**Published:** 2024-01-09

**Authors:** Michelle Symons, Heidi Vandebosch, Karolien Poels

**Affiliations:** Department of Communication Studies, University of Antwerp, Antwerp, Belgium

**Keywords:** *Humorous intervention messages.*, *Cognitive Fatigue.*, *Brisk Walking Intentions.*, *Self-Control.*, *Smartphone Application.*

## Abstract

**Background:**

A well-studied internal barrier to regular physical activity, and more specifically brisk walking, is cognitive fatigue. However, thus far little research examined how cognitively fatigued individuals can be motivated to exercise, more specifically to engage in brisk walking. This study investigates whether humorous intervention messages might be an effective strategy to motivate cognitively fatigued individuals to brisk walk, and through which underlying processes.

**Methods:**

An online experiment was performed in which variation in cognitive fatigue was induced through mental arithmetic questions. Afterwards, participants (*n* = 250) recruited through Prolific, randomly received either humorous or non-humorous intervention messages related to brisk walking. Potential mediators of the relations between physical activity, humour and cognitive fatigue were measured, were self-efficacy, self-control, and motivation.

**Results:**

First, regression analyses confirmed that cognitive fatigue negatively influences brisk walking intentions and that the perceived humour of the intervention messages moderated this relationship. Second, results showed that self-control and self-efficacy are mediators explaining the relationship between cognitive fatigue and brisk walking intentions. Lastly, this study found that perceived humour of the intervention messages moderated the relationship between cognitive fatigue and self-control, indicating that perceptions of self-control were positively changed after receiving messages that were perceived as humorous compared to messages that were not perceived as humorous, subsequently increasing brisk walking intentions.

**Conclusions:**

This study is the first to unravel the underlying relationship between humorous intervention messages and brisk walking intentions through positive changes in perceptions of self-control within a cognitively fatigued sample. Results of this study suggest that existing smartphone applications monitoring and promoting brisk walking should integrate tailored message strategies within their cues to brisk walk by implementing humour as a strategy to motivate users when they are cognitively fatigued.

**Supplementary Information:**

The online version contains supplementary material available at 10.1186/s12889-023-17464-z.

## Introduction

Physical inactivity is a worldwide health problem, with almost 25% of the overall population being physically inactive [1]. A combination of external barriers (such as lack of support, resources, and time…) and internal barriers (such as lack of energy, confidence, and motivation…) often hinder individuals to be active regularly, such as taking a recommended 30 min brisk walk each day [2–4]. Brisk walking can be defined as a moderate-intensity activity where most adults walk at a pace of three to four mph [5].

Until recently, most studies have only targeted external barriers when trying to increase physical activity levels, such as brisk walking behaviours [6]. For instance, to overcome barriers like ‘*I don’t have time to brisk walk*’ the studies of Lin and colleagues [7, 8] used data points including GPS-location and electronic diary appointments to send a message motivating the user to engage in brisk walking – thus fitted into the schedule of the user. However, the targeting of internal barriers has been mostly overlooked within physical activity interventions overall, and thus also within brisk walking interventions [9–11]. Hence, this paper focuses on (overcoming) a well-studied internal barrier to brisk walking, and by extension physical exercise overall, namely cognitive fatigue [12–14].

Cognitive fatigue can be understood as an intellectual failure to complete mental tasks, which occurs after performing mentally demanding tasks for a prolonged time, for example when feeling mentally exhausted after extensive work-related tasks, such as reading, writing, thinking, etc. These feelings of cognitive fatigue are known to impede engagement in physically active behaviours by influencing the intended willingness to exercise and exercise intensity, which in turn lowers the actual exercise performance [15, 16]. However, current research has mostly examined the relationship between cognitive fatigue and vigorous-intensity physical activities, like running and cycling [12, 15, 17–19]. Therefore, within this study we want to examine if cognitive fatigue is also a perceived barrier for moderate-intensity activities, specifically brisk walking.

Further, current smartphone-based interventions that try to increase physical activity levels by focussing on brisk walking behaviours (e.g., measuring steps) mostly rely exclusively on external data points, such as location, in combination with predetermined activity goals, like steps [6]. Subsequently, these health applications use strategies and techniques that are tailored to these external factors, including behavioural change techniques, for instance, goal-setting [20]. Although advances in technology are ensuring messages are increasingly personalised and tailored to the user, the messages in themselves often remain similar to each other and rather monotonous by often relying on the same techniques, e.g., “*Only 100 steps until you’ve reached your goal*”. Therefore, it would be beneficial for the user if smartphone-based interventions would consider more specific (internal) barriers, such as cognitive fatigue, and integrate specific solutions within their intervention messages trying to overcome these barriers, thereby using more innovative strategies, such as humour [9, 21].

Humour has been found to have the ability to change perceptions of cognitive fatigue to baseline levels (i.e., a cognitively vital state) [22]. In addition, it is found that humour can work as an emotion regulator by down-regulating negative and up-regulating positive emotions when feeling cognitively fatigued [23]. Therefore, this paper contributes to the literature by examining if messages that are perceived as humorous are more effective in motivating cognitively fatigued individuals to brisk walk compared to non-humorous messages. In addition, converting from a negative cognitive state to a more positive state has a positive influence on several determinants related to physical activities like brisk walking, such as self-efficacy, self-control, and motivation [24–26]. At the same time, these determinants are positively related to exercise adherence [24–26] and negatively related to cognitive fatigue [22, 27, 28]. More specifically, humour has been found to increase levels of self-efficacy [29], levels of self-control [30], and motivation levels [31]. As such, this research aims to examine if and how these determinants mediate the relationship between cognitive fatigue and brisk walking behaviours, and, in addition, whether humour (can) influence these determinants. In order to maximise the success of future interventions, it is crucial to determine if humour simply moderates the relationship between cognitive fatigue and brisk walking or whether there is more to it.

**Fig. 1:**
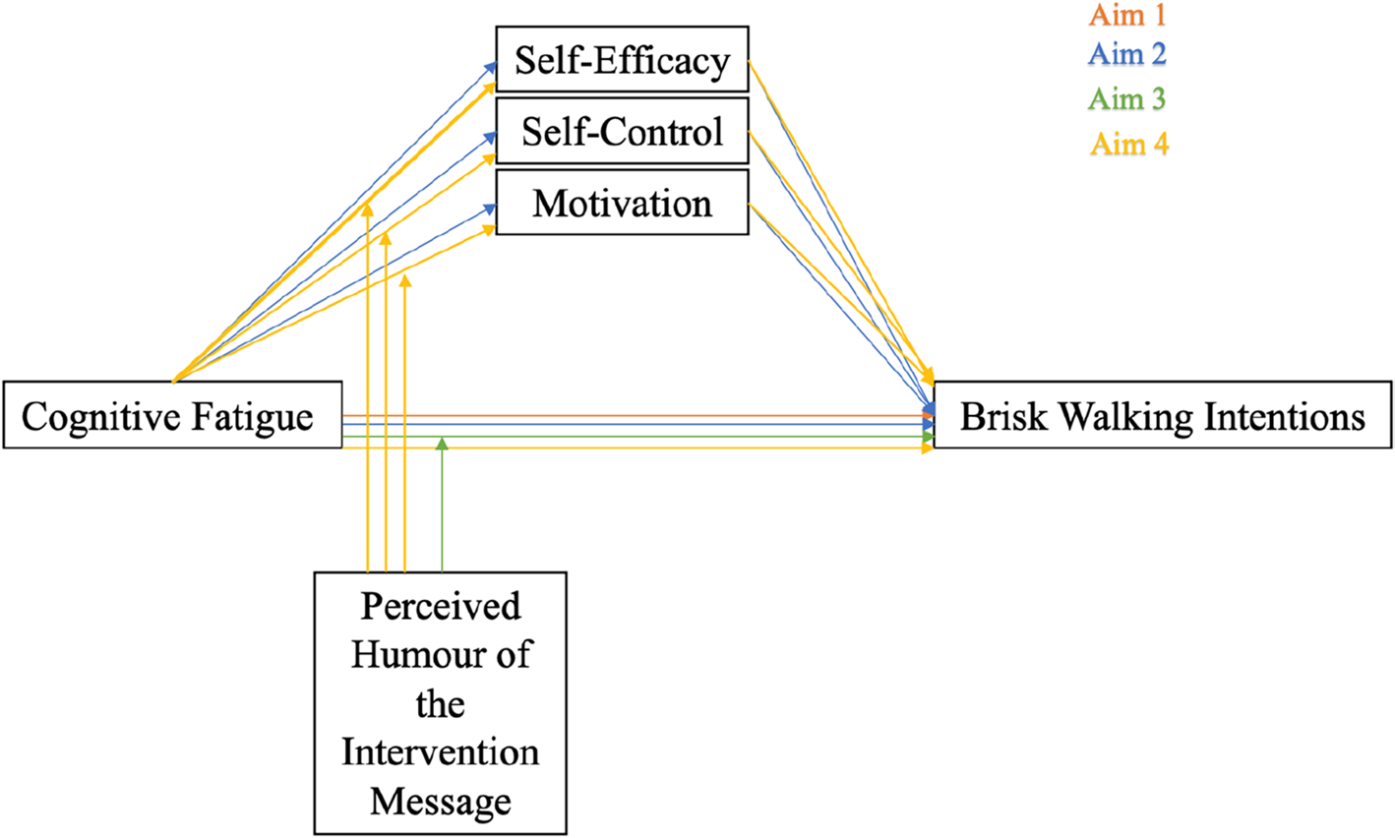
Overview of the study variables

Thus, the current study considers four objectives (see Fig. 1). The main objective is to examine whether feelings of cognitive fatigue negatively predict brisk walking intentions. The second objective is to unravel the process through which this happens; is it by (negatively) influencing self-efficacy, self-control, and/or motivation? Thirdly, we want to understand if intervention messages that are perceived as humorous moderate the relationship between feelings of cognitive fatigue and brisk walking intentions. And lastly, if a mediation of self-efficacy, self-control and/or motivation exists, we will test whether the perceived humour of the intervention messages promoting brisk walking behaviours moderates the aforementioned mediations.

## Literature review

### Physical (in)activity

Physical activity, defined as “any bodily movement produced by the contraction of skeletal muscle that increases energy expenditure above a basal level”, comes in three levels: light, moderate, and vigorous [1, 32]. Recommendations include engaging in light activities whenever possible and aiming for 150 minutes of moderate activity, or 75 minutes of vigorous activity to maintain a healthy lifestyle [1]. Failing to meet these guidelines constitutes inactivity, a concerning trend with a 5 % increase from 2001 to 2016 [1]. Regular physical activity significantly reduces the risk of chronic diseases and promotes mental well-being [33, 34]. Despite these benefits, physical inactivity remains a major public health issue, ranking as the fourth leading cause of mortality, responsible for about 3.2 million deaths annually [35, 36]. Hence, there remains a critical need for effective interventions to promote physical activity for individual and population health.

### Cognitive fatigue

As already mentioned in the introduction, the literature states that cognitive fatigue negatively influences exercising intentions and behaviours. For instance, the study of Martin and Bray [37] exposed participants to a cognitively fatiguing task during a lab experiment, afterwards exposing them to a 10-minute bicycle task. Cognitively fatigued participants were found to exert lower levels of exercise during the cycling task and were found to be planning to exert less effort during an upcoming cycling task, compared to a control condition. Similarly, the study of Brown and Bay [19] exposed participants to a cognitively demanding task whereafter they were examined about their intended physical effort and their total work for a 30-min self-paced exercise task. Findings showed significantly greater cognitive fatigue, significant reductions in intended physical exertion and significantly less work performed during the exercise session compared to the control condition. As such, it is evident addressing cognitive fatigue is needed to help the public become more active.

Further, cognitive fatigue often prompts a need for recovery, which can be fulfilled in two ways: passive activities like watching television or dynamic activities like brisk walking [38–41]. Research suggests that physical activity can alleviate cognitive fatigue and reduce its overall levels [42, 43]. However, individuals often prefer passive activities, highlighting the need to motivate cognitively fatigued individuals to engage in exercise [14].

As there exist several forms of physical activities, within this paper we will focus on brisk walking behaviours. Brisk walking is found to produce substantial health benefits, and beyond that, it is an easy-to-perform activity for which you do not need any equipment, expertise, preparations, or knowledge [5]. Brisk walking is also used in other smartphone interventions that try to enhance physical activity levels [10, 11], and also when feeling cognitively fatigued [44]. Based on this knowledge, we hypothesise the following relationship:

*H1: Cognitive fatigue will negatively predict brisk walking intentions.*

### Perceived humour of the intervention messages that promote brisk walking behaviours

There are reasons to assume that humorous messages can be especially persuasive in motivating cognitively fatigued individuals. Humorous messages can elicit a positive mood [45], which is found to alter individuals’ evaluations of their experienced cognitive fatigue [22], and, according to the meta-analysis of Cameron and colleagues [46], positive mood interventions engender more favourable outcome expectancies concerning physical activity. Additionally, research shows that altering people’s perceptions of cognitive fatigue by inducing a positive state counteracts the effects of cognitive fatigue and leads to replenishment [47, 48]. For instance, Egan and Hirt [49] found that when participants are subject to a cognitively fatiguing task and are afterward induced to a positive state, their perceptions of cognitive fatigue were significantly lower than those of participants who were induced to a negative state. Moreover, the level of perceived cognitive fatigue of participants who experienced the positive state did not differ significantly from participants not exposed to a fatiguing cognitive task (control condition). Additionally, when a user’s cognitive capacity is reduced due to cognitive fatigue [22], less effort is invested in seeing, reading, and interpreting messages [50]. As a result, an easier, more surprising message might be more effective in enhancing behaviours than a more serious or informative message [51].

Although studies often compare humorous and non-humorous messages, literature shows that perceived humour should also be measured, as a humorous message might not be perceived as humorous by everyone [52]. Subsequently, studies show that perceived humour plays a large role in determining whether the humorous message is effective or not [53]. For instance, Skalski and colleagues [54], did not find differences between humorous and non-humorous messages in anti-binge drinking ads on reactance. However, perceived humour was negatively associated with reactance. The same was true for the study of Pariera [29], who did an eight-week intervention on parent-child sexual communication and found no difference between humorous and non-humorous messages but stated that perceived humour was positively associated with self-efficacy. Therefore, this study focuses on perceived humour instead of solely examining humorous vs. non-humorous messages. Departing from the findings that messages that are perceived as humorous are more effective in health interventions [29, 54], and are able to alter individuals’ evaluation of their experienced cognitive fatigue [22, 49], we formulate the following hypothesis:

H2: Perceived humour of the intervention messages that promote brisk walking behaviours will moderate the relationship between cognitive fatigue and brisk walking intentions so that humorous messages are more effective in promoting brisk walking intentions in fatigued individuals compared to non-fatigued individuals*.*

### Self-efficacy

Self-efficacy is derived from social cognitive theory and can be described as “*the believes an individual has in their capacity to perform a behaviour*” [55, 56]. The discussion paper of Lee and Buchner [24] argues that self-efficacy is strongly connected with the amount of physical activity individuals undertake. Similarly, the study of Depenbusch and colleagues [57] found that lower levels of self-efficacy decreased patients’ intention and their likelihood to engage in physical exercise. Also, Marcus & Owen [58] report that self-efficacy levels among healthy individuals significantly differ depending on the stage of physical activity change they are in, with significantly higher levels in the regularly active stage. Further, the review analysis of Williams and French [59] looked at 27 unique studies among healthy adults. They concluded that if a significant effect of an intervention technique was found on change in self-efficacy, it tended to be associated with a positive change in effect size for physical activity. Furthermore, the study of Hejazizadeh [27] examined the relationship between fatigue (physical and cognitive) and self-efficacy amongst multiple sclerosis patients and found that higher mean scores of fatigue resulted in lower mean scores of self-efficacy. In addition, the study of Haas [60] investigated 73 women that were treated for breast cancer and suggested that cancer-related fatigue (seen primarily as a psychological or thus cognitive form of fatigue) is a potential barrier to engaging in physical activity, mediated by self-efficacy, subsequently improving quality of life. The participants had to fill out a questionnaire and structural equation modelling showed the relations to be significant. Although these findings are examined in patient populations, research overall argues that the implementation of self-efficacy enhancing techniques in intervention programs may help to overcome psychological barriers, such as cognitive fatigue, for increasing physical activity [60–62]. Therefore, we hypothesise self-efficacy to be the first out of three potential mediators:

H3_a_: Self-efficacy will mediate the relationship between cognitive fatigue and brisk walking intentions*.*

As already mentioned before, the study of Pariera [29] found no difference between humorous and non-humorous messages on self-efficacy but did find that *perceived* humour was positively associated with self-efficacy related to parent-child sexual communication. Similarly, Bleakley and colleagues [63] did a study with three experimental conditions (humour, fear, and nurturance) and one control condition, examining the effect of different emotional appeals on the intention to drink sugar-sweetened beverages. The results showed that all ads increased levels of self-efficacy compared to the control ad. Overall, when it comes to the perceived humour of a message, a positive trend towards a positive relation between humour and self-efficacy can be found [29, 54]. In combination with the negative relation between cognitive fatigue and self-efficacy [27] and the interaction effects between cognitive fatigue and humour [30], we hypothesise the following relation:

H3_b_: Perceived humour of the intervention messages that promote brisk walking behaviours will moderate the relationship between cognitive fatigue and self-efficacy so that perceptions of self-efficacy will be positively changed after receiving messages that are perceived as humorous compared to messages that are not perceived as humorous*.*

### Self-control

Self-controlled behaviour refers to “*voluntary actions in which individuals engage to advance personally valued longer-term goals despite conflicting urges that are more potent in the moment*” [64] (e.g., exercising rather than watching television). The study of Finne and colleagues [65] found that high levels of self-control promote the implementation of short-term exercise intentions. The authors studied exercise intentions and levels of self-control amongst 259 participants for 13 weeks. They found that weekly exercise intentions were only met when self-control was high. Furthermore, within the meta-analysis of Clarkson and colleagues [22] the relationship between cognitive fatigue and self-control was examined based on 16 studies. The data support a significant impact on individuals’ perceptions of cognitive fatigue for subsequent self-control exertion. As cognitive fatigue negatively predicts exercise intention and self-control [16, 22], and self-control is positively related to exercise intention [65], we predict the following relationship between these three concepts, with self-control being the second out of three possible mediators:

H4_a_: Self-control will mediate the relationship between cognitive fatigue and brisk walking intentions*.*

Concerning the effects of humorous messages, the experimental study of Cheng and Wang [30] found a causal impact of humour on persistence behaviour, after inducing participants to a cognitively fatigued state. First, participants had to cross out the letter “e” of an essay to induce a fatigued state, whereafter they were exposed to either a humorous or a sad video. The authors found a causal effect between humour and persistence with replenishing effects of cognitive fatigue when watching a humorous video. This indicates an interaction between humour and cognitive fatigue on persistence. Further, they specify that this causal relationship is mediated by perceived humour. Although this study did not explicitly examine self-control, persistence towards a goal is only possible when exerting sufficient self-control [66]. As cognitive fatigue negatively influences self-control [22], and humour directly affects self-control [30], and there has been found an interaction between cognitive fatigue and humour [30], we suggest the following:

H4_b_: Perceived humour of the intervention messages that promote brisk walking behaviours will moderate the relationship between cognitive fatigue and self-control so that perceptions of self-control will be positively changed after receiving messages that are perceived as humorous compared to messages that are not perceived as humorous*.*

### Motivation

Motivation is defined as “*a driving force or forces responsible for initiation, persistence, direction and vigour of goal-directed behaviour*” [67]. Motivation is found to positively influence physical activity intention [68] and is described as a central determinant in many programs trying to enhance physical activity behaviours [69]. The randomized crossover study of Marcora and colleagues [12] shows that cognitive fatigue can negatively affect motivation for subsequent performance. Also, the study of Boksem and colleagues [70] found that fatigued participants – who had to complete a two-hour task to induce fatigue – could monitor their actions more adequately after motivating them to perform the behaviour. However, a more recent systematic meta-analytic review (consisting of eight papers) by McMorris and colleagues [28] found rather mixed results considering the relationship between cognitive fatigue and motivation. Therefore, the authors suggest that more studies are needed. Based on the knowledge that motivation positively influences physical activity intention and might be negatively influenced by cognitive fatigue, we predict motivation being the third out of three possible mediators, suggesting:

H5_a_: Motivation will mediate the relationship between cognitive fatigue and brisk walking intentions*.*

The relationship between humour and motivation is already well-established in the context of health care and education [31]. It has been demonstrated that the use of humour reduces anxiety, enhances participation, and increases motivation when directly linked to the central message one is trying to bring [71, 72]. It is found that motivation does not directly cause learning but creates an environment that promotes learning, which could also be true in the context of promoting physical exercise. However, when the humour used is not understood or perceived as inappropriate, thus not perceived as humorous, motivation will decrease [73]. Based on the knowledge that perceived humour is positively related to motivation [31], in combination with the negative relation between cognitive fatigue and motivation [12], and the interaction effects between cognitive fatigue and humour [30], we suggest:

H5_b_: Perceived humour of the intervention messages that promote brisk walking behaviours will moderate the relationship between cognitive fatigue and motivation so that perceptions of motivation will be positively changed after receiving messages that are perceived as humorous compared to messages that are not perceived as humorous*.*

## Methods

### Participants

This study consisted of an online experiment with a 2 (fatigue: fatigue manipulation, no manipulation) by 2 (activity message: humorous, non–humorous) between-subjects design, administered through Qualtrics. An a priori power analysis using G*Power 3.1 [74] for linear multiple regression: fixed model, R^2^ deviation from zero considering eight predictors (fatigue, message, self-efficacy (T1 and T2), self-control (T1 and T2), motivation (T1 and T2)), indicated that with 160 participants an effect size of 0.15 could be captured with 95% of power [75].

Participants were recruited through Prolific, a database with participants for online research [76]. Before making the study available on Prolific, inclusion criteria were added to reach a more coherent sample. As such, participants had to be citizens of the United Kingdom, fluent in English, not have a student status, and experience similar work conditions (working a 9 to 5 job, remotely and/or at the office). Additionally, this study examined adults aged between 25 and 45 years old as younger workers are more prone to burnout resulting from prolonged fatigue [77] Lastly, we indicated on the Prolific website we were looking for a balanced sample (50% males, 50% females).

Participants were asked to complete a 15-minute online experiment and were compensated £2.40 per participation. The final sample size consisted of 250 individuals. Informed consent was obtained from all participants and study procedures were approved by the Independent Ethical Advisory commission for research in Social and Human Sciences (EA SHW_18_81).

### Procedure

**Fig. 2:**
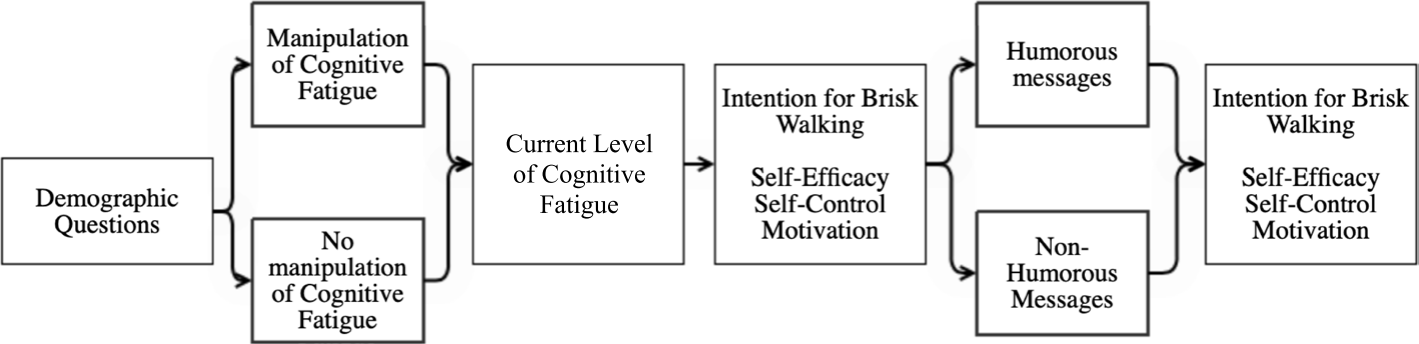
Procedure

To better understand the procedure of this online experiment, consult Fig. 2. When entering the online experiment, participants had to answer some demographic questions to be able to describe the study sample. In addition, physical activity levels and cognitive effort after a regular day at work were questioned. Afterwards, participants were randomly assigned to one of two conditions: either they had to solve math problems to ensure that some of them felt cognitive fatigue, or they immediately moved to the next part of the study [78]. Next, all participants had to consider their current cognitive fatigue measured from one to seven. Thereafter, they were asked to answer their intention to go for a brisk walk at that moment (7-point Likert scale), measures of self-efficacy to brisk walk, self-control to brisk walk and motivation to brisk walk. Subsequently, participants were either exposed to a sequence of nine humorous or nine non-humorous messages motivating individuals to brisk walk (see Additional file 1). Humorous messages consisted of cat pictures with congruent text, as cats are found to easily embody human feelings and behaviours in a humoristic fashion accessible to a broad audience [79]. The non-humorous messages consisted of the same text but displayed a neutral white sports shoe instead of cats [44]. Lastly, participants’ likeliness to go for a brisk walk, levels of self-efficacy to brisk walk, self-control to brisk walk and motivation to brisk walk were asked again after seeing the messages.

### Measures

#### Demographic questions

Participants were asked: “*What gender do you identify most with?*” (male or female); “*What ethnic group do you belong to?”* (Black, White, Asian, mixed, prefer not to answer, or other); “*What is your year of birth?”*(open question); *“What is your highest qualification?”* (lower than secondary education, secondary education/equivalent, academic, or professional bachelor/advanced bachelor/equivalent, master/post-master/equivalent, or PhD); *“What is your employment status*?” (full-time 100% or more, more than half time, half-time, less than half-time, or do not work).

#### Physical activity

Participants were asked how much time they spent seated on an average working day (more than 50%, approximately 50%, less than 50%), how many days per week they went for a brisk walk^1^ during the last 3 months (times per week ranging from one to seven), how many minutes per day they went for a brisk walk in the last 3 months (open question), and how many minutes on average they spent on brisk walking the preceding week (open question). Similar questions to examine exercise behaviours were asked by Gillebaart & Adriaanse [80].

#### Cognitive effort after work

The question “*Overall, how mentally exhausted do you feel at the end of your working day?*” tried to understand how mentally exhausted participants felt after a regular working day. Participants could indicate their answers with a slider from one to seven.

#### Cognitive fatigue (independent variable)

Before (T1) and after the manipulation (T2_a_), or just after the demographic questions when there was no manipulation (T2_b_), participants were asked to indicate their current level of cognitive fatigue^2^ with a slider ranging from 1 (= not cognitively fatigued at all) to 7 (= extremely cognitively fatigued) [81]. This resulted in a continuous variable where both cognitive fatigue levels after the manipulation (T2_a_) and cognitive fatigue levels without manipulation (T2_b_) were measured.

#### Intention to brisk walk (dependent variable)

Participants were asked the following, before and after the humorous or non-humorous messages were shown: “*Considering your current mental state, and only your mental state, how likely is it that you would go for a brisk walk at the moment?*”. Their answers varied from extremely unlikely to extremely likely on a 7-point Likert scale [82].

#### Self-efficacy (mediator)

Based on the study of Hagger and colleagues [83] and the book of Brug and colleagues [84], items to measure self-efficacy before and after the messages were: “*It is easy for me to go for a brisk walk*”, “*It is hard for me to go for a brisk walk*”, “*I am confident that I can go for a brisk walk*”, and “*I am able to go for a brisk walk*”. Responses were given on a 5-point Likert scale (1 = strongly disagree to 5 = strongly agree) and the internal consistency of this scale was satisfactory (α_T1_ = 0.91; α _T2_ = 0.89).

#### Self-control (mediator)

The 5-Item Scale for Measuring Momentaneous Self-Control Capacity (SMS-5) was used to measure self-control before and after viewing the activity messages, considering participants’ current state. The items consisted of “*I feel drained”, “I feel calm and rational*”, “*I feel lazy*”; “*I feel sharp and focused*”, and “*I feel like my willpower is gone*” [85]. Participants had to answer if they agreed or disagreed with each statement on a 7-point Likert scale ranging from strongly disagree to strongly agree. The scale proved reliable with a Cronbach’s α_T1_ = 0.85 and α_T2_ = 0.83.

#### Motivation (mediator)

Motivation to exercise was measured with the Self-Determination Motivation Scale for Exercise-2 (SMSE-2) using the intrinsic motivation and amotivation subscales. The intrinsic motivation subscale asked participants how much they agreed with the following statements considering their current state: *“Brisk walking itself is fun”, “I can focus on my routine while going for a brisk walk”, “Brisk walking is an activity that makes me feel satisfied”, “It gives me pleasure to learn about brisk walking”,* whereas the amotivation subscale items were: *“I do not know why I brisk walk”, “I cannot see why I should brisk walk”, “I do not have any reason to brisk walk”* [86]. The latter three items were recoded, and all items together resulted in a reliable scale with a Cronbach’s α_T1_ = 0.84 and α_T2_ = 0.85. All items were measured on a 5 point-Likert scale.

#### Perceived humour (moderator)

Participants had to indicate if they perceived the messages as not funny – funny, not amusing – amusing, not entertaining – entertaining, not humoristic – humoristic [87]. Cronbach’s proved the scale to be reliable: α = 0.98.

### Statistical analyses

In the result section, we will present the findings of our analyses, which were all conducted using SPSS version 28. Additionally, we employed the PROCESS macro developed by Hayes [88] to explore mediation and moderation effects in our study. Our analyses will be divided into several subsections, each addressing specific research objectives and hypotheses. First, descriptive statistics and Pearson correlations, as well as an overview of the manipulation checks will be discussed. The following part consists of hypothesis testing, organised by hypothesis.

In order to exert more control over individuals’ states and to make sure there was enough variation in cognitive fatigue measures, we opted to use a manipulation. Manipulations of cognitive fatigue are common, however, there is not one method that is used as the preferred option [78, 89, 90]. Based on a comparison of different papers and the feasibility of the manipulation within an online experimental context, we decided to use mathematical additions and subtractions to increase levels of cognitive fatigue [91]. One group of participants randomly got exposed to the manipulation and had to report current levels of cognitive fatigue before and after the manipulation. The other group (no manipulation) only had to report current levels of cognitive fatigue. Participants were asked not to use any aids in solving the questions, additionally, each addition or subtraction was only shown for 10 seconds and thereafter the next addition or subtraction was shown automatically. To assess whether the manipulation of cognitive fatigue was effective or not, we relied on a one-way ANOVA. As such, we wanted to understand if baseline levels of cognitive fatigue differed between the two groups. Therefore, a One-Way ANOVA with group (0 = no manipulation; 1 = manipulation) as independent variable and baseline levels of cognitive fatigue as dependent variable was performed. Next, a Repeated Measures ANOVA with the first measure of cognitive fatigue (T1 - before manipulation) and the second measure of cognitive fatigue (T2a - after manipulation) as dependent variables was performed to understand whether there was an increase in cognitive fatigue after the manipulation or not. Lastly, we wanted to know if there was a significant difference between both groups after the manipulation. A One-Way ANOVA with group (0 = no manipulation; 1 = manipulation) as independent variable and levels of cognitive fatigue as dependent variable was performed. After randomly exposing participants to either the humorous messages or the non-humorous messages, we asked them if they perceived the messages as humorous or not based on a perceived humour scale used by Nabi and colleagues [87]. A One-Way ANOVA with the condition (0 = receiving non-humorous messages, and 1 = receiving humorous messages) as independent variable and perceived humour as dependent variable was performed to understand if individuals subjected to the humorous messages also perceived the messages as significantly more humorous than the individuals in the non-humorous condition. In order to answer the first hypothesis, that cognitive fatigue negatively predicts brisk walking intentions, we performed a linear regression with cognitive fatigue as independent variable, and brisk walking intentions (T1– before viewing messages) as dependent variable (assumptions of independency, multicollinearity, linearity, normality, and homoscedasticity were met).

Next, we wanted to understand if the perceived humour of the intervention messages that promote brisk walking behaviours moderates the relationship between cognitive fatigue and brisk walking intentions so that humorous messages are more effective in promoting brisk walking intentions to fatigued individuals compared to non-fatigued individuals. Therefore, a moderation analysis was performed with cognitive fatigue as independent variable, intentions to brisk walk (T2 – after receiving messages) as dependent variable, perceived humour as moderator and intention to walk (T1) as covariate (Model 1–10,000 bootstrap intervals - BC 95% confidence intervals).

Further, to test hypotheses H3a, H4a, and H5a and thus understand if self-efficacy, self-control, and motivation mediate the relationship between cognitive fatigue and intention to brisk walk (T1), we looked at mediation effects with PROCESS [88] (model 4–10,000 bootstrap intervals - BC 95% confidence intervals). Cognitive fatigue was used as independent variable, brisk walking intentions (T1) as dependent variable, and self-efficacy (T1), self-control (T1), and motivation (T1) as mediators.

To understand if the (humorous) messages were effective in enhancing levels of self-efficacy (T2), self-control (T2), and motivation (T2) and thereby increase brisk walking intentions (T2) whilst experiencing cognitive fatigue (H3b, H4b and H5b), we calculated the difference between self-efficacy (T1) and self-efficacy (T2), self-control (T1) and self-control (T2), motivation (T1) and motivation (T2), and brisk walking intentions (T1) and brisk walking intentions (T2). This results in four new delta variables, namely self-efficacy (ΔT), self-control (ΔT), motivation (ΔT) and brisk walking intentions (ΔT). These delta variables are used because only in this way the effectiveness of the moderator (i.e., perceived humour of the intervention messages) can be measured with impact, namely understanding the change in self-efficacy, self-control, motivation, and brisk walking intentions, following viewing either humorous or non-humorous messages. Using these delta variables, we tested hypotheses H3b, H4b and H5b, looking at moderated mediation of perceived humour of the intervention messages that promote brisk walking behaviours on the mediation of self-efficacy, self-control, and motivation with PROCESS (model 7–10,000 bootstrap intervals - BC 95% confidence intervals). Cognitive fatigue was used as independent variable, brisk walking intentions (ΔT) as dependent variable, self-efficacy (ΔT), self-control (ΔT), and motivation (ΔT) as mediators and perceived humour as moderator.

## Results

### Descriptive statistics

Participants (*n* = 250) were on average 36 years old (Mage = 35.62; SD = 5.89), 50% of the sample were males and 50% were females. Most individuals identified as ‘white’ when asked about ethnic grouping (85.2%) and the highest level of education was mostly academic bachelor, professional bachelor or equivalent (53.6%). Most participants indicated to work full-time or more (82.8%), are sitting equal to or more than 50% of the time at work (87.6%) and are brisk walking for about 135 minutes per week (3 days multiplied by 45 minutes per day). Considering the guidelines of the World Health Organization, this sample can be viewed as sedentary and inactive [1, 3]. For more details concerning the characteristics of the sample, see Table 1.

**Table 1:** Characteristics of the sample

**Continuous Study Variables**	*M*	*SD*
Age (years)	35.6	5.9
Mental exhaustion at the end of the working day (0–7)	4.5	1.5
How many days per week do you go for a brisk walk (past 3 weeks) (0–7)	3.3	1.9
How many minutes per day do you go for a brisk walk (past 3 weeks)	45.2	92.1
How many minutes did you walk at brisk pace during the week preceding this day	103.2	112.5
**Categorical Study Variables**	***N***	***%***
**Gender**		
Men	125	50.0
Women	125	50.0
**Ethnic Grouping**		
Black	12	4.8
White	213	85.2
Asian	18	7.2
Mixed	6	2.4
Prefer not to answer	1	0.4
**Education**		
Lower than secondary education	1	0.4
Secondary Education or equivalent	63	25.2
Academic bachelor or professional bachelor or equivalent	134	53.6
Academic maser or post-master or equivalent	49	19.6
PhD	3	1.2
**Employment Status**		
Employed full-time (100% or more)	207	82.8
Employed more than part-time (> 50%)	20	8.0
Employed part-time (50%)	14	5.6
Employed less than part-time (< 50%)	8	3.2
Not employed	1	0.4
**Perceived Sitting Time at Work**		
More than 50% of the time	177	70.8
About 50% of the time	42	16.8
Less than 50% of the time	31	12.4

### Pearson correlations

Correlations between cognitive fatigue, perceived humour and all dependent variables can be found in Table 2. All dependent variables that significantly correlate with cognitive fatigue, indicate a very weak or weak negative correlation with cognitive fatigue, except for self-control (T1 and T2) where the correlation is also negative but moderate. All other significant correlations between variables are positive. Strong and very strong correlations are found between time one and time two measurements, such as the correlation between self-control (T1) and self-control (T2) (*r* = .83, *p* < .001). Also, there is a strong correlation between brisk walking intentions (T1) and self-efficacy (T1 and T2) (*r*_*T1*_ = .72, *p* < .05; *r*_*T2*_ = .65, *p* < .05).

**Table 2:** Pearson correlations

	1	2	3	4	5	6	7	8	9	10
1 Cognitive Fatigue	1									
2 Perceived Humour	−.08	1								
3 Self-Efficacy (T1)	−.30**	.06	1							
4 Self-Efficacy (T2)	−.20**	.17**	.78**	1						
5 Self-Control (T1)	−.58**	.14*	.48**	.44**	1					
6 Self-Control (T2)	−.42**	.18**	.46**	.50**	.83**	1				
7 Motivation (T1)	−.04	.10	.45**	.51**	.26**	.28**	1			
8 Motivation (T2)	−.03	.23**	.42**	.54**	.22**	.34**	.87**	1		
9 Brisk Walking Intentions (T1)	−.18**	.12*	.72**	.65**	.44**	.36**	.56**	.59**	1	
10 Brisk Walking Intentions (T2)	.014	.38**	.45**	.58**	.23**	.32**	.56**	.58**	.58**	1

* Correlation is significant at the 0.05 level (1-tailed)

** Correlation is significant at the 0.01 level (1-tailed)

### Manipulations

#### Cognitive fatigue

The results of a One-Way ANOVA show that baseline levels of cognitive fatigue were not significantly different between both the manipulated and non-manipulated group (*F*(1,248) = .15, *p* = 0.696, *η*_*p*_^*2*^ = 0.00). Next, Repeated Measures ANOVA resulted in significant differences between time one and time two (*F*(1,121) = 54.81, *p* < 0.000, *η*_*p*_^*2*^ = 0.31). Additionally, mean scores revealed that cognitive fatigue increased from time one to time two (*M*_*T1*_ = 3.73, *SD*_*T1*_ = 1.45 and *M*_*T2*_ = 4.44; *SD*_*T2*_ = 1.53). The results of the last One-Way ANOVA show that there is a significant difference between the two groups, where the manipulated group experienced more cognitive fatigue compared to the non-manipulated group (*F*(1, 247) = 11.32, *p* < 0.001, *η*_*p*_^*2*^ = 0.04; *M*_*Manipulation*_ = 4.44, *SD =* 1.53; *M*_*No Manipulation*_ = 3.81, *SD* = 1.43). These results indicate that the manipulation of cognitive fatigue succeeded. In the continuation of the statistical analyses, the effect of cognitive fatigue on walking intentions is examined using self-reported cognitive fatigue as a continuous variable.

#### (Humorous) messages

A One-Way ANOVA showed that the individuals subjected to the humorous messages also perceived the messages as significantly more humorous than the individuals in the non-humorous condition (*F*(1, 248) = 93.76, *p* < 0.001, *η*_*p*_^*2*^ = 0.27; *M*_*humour*_ = 5.29, *SD*_*humour*_ = 1.74; *M*_*non-humor*_ = 3.20, *SD*_*non-humor*_ = 1.67). As already mentioned in the literature part that perceiving something as humorous or non-humorous is a rather individual experience [52], we will use the continuous variable ‘perception of humour’ in all upcoming analyses.

### Hypothesis testing

#### Hypothesis 1

**Fig. 3:**
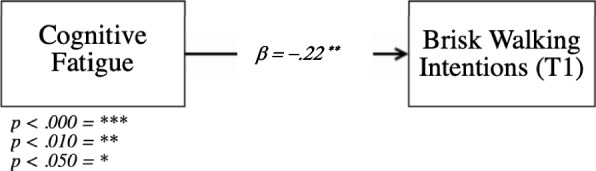
Visual presentation of H1

The overall regression (Fig. 3) was statistically significant (R^2^ = 0.03, *F*(1, 247) = 7.86, *p* = 0.005). Individuals’ current levels of cognitive fatigue are a significant and negative predictor for brisk walking intentions (*β* = −.22, *SE* = .08, *p* = 0.005), thus accepting our first hypothesis.

#### Hypothesis 2

**Fig. 4:**
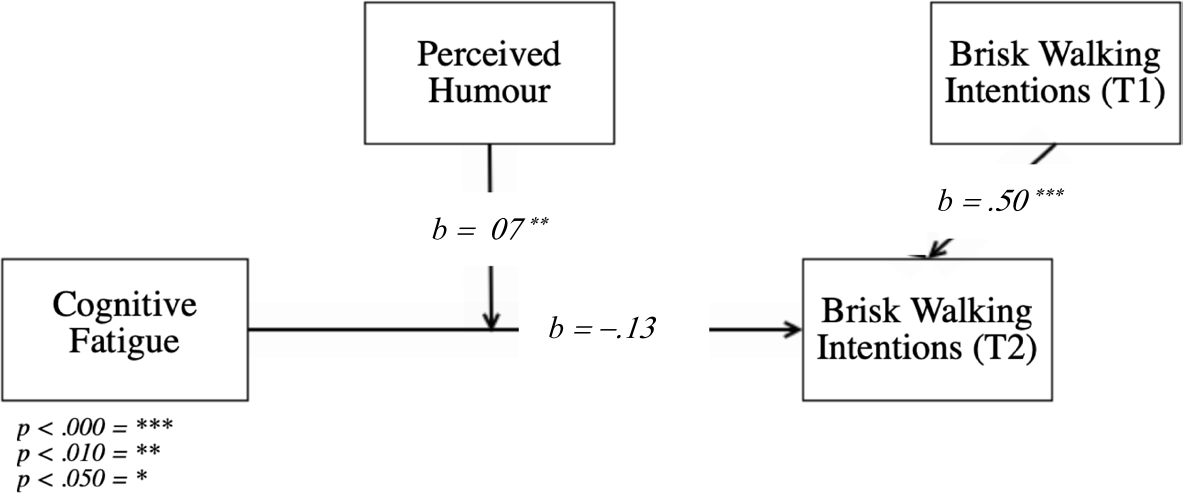
Visual presentation of H2

The overall moderation model (Fig. 4) was significant (R^2^ = 0.47, *F*(4, 244) = 55.13, *p* < 0.001). Further, the model showed that the covariate intention to walk (T1) is a significant predictor of intentions to brisk walk (T2) (*t*(244) = 11.72, *p* < .001, *b* = .50, *SE* = .04).

Additionally, the direct effects of cognitive fatigue (*t*(244) = − 1.07, *p* = .286, *b* = −.13, *SE* = .12) and perceived humour (*t*(244) = .21, *p* = .831, *b* = .02, *SE* = .12) on brisk walking intentions (T2) were insignificant, but the interaction between cognitive fatigue and perceived humour was positive and significant (*t*(244) = 2.67, *p* = .008, *b* = .07, *SE* = .03), which indicates a moderation of perceived humour on the relationship between cognitive fatigue and brisk walking intentions (T2).

More specifically, based on the Johnson-Neyman significance regions, we can understand that only when perceived humour scores at least 3.63 out of seven points, perceived humour and cognitive fatigue are significantly related (*t*(244) = 1.97, *p* = .050, *b* = .11, *SE* = .06), and thus only when a message is perceived as humorous enough (= starting from 3.63 out of seven), an interaction between perceived humour and cognitive fatigue takes place. As perceived humour increases, the relationship between cognitive fatigue and perceived humour becomes more positive, with seven being the highest possible value for perceived humour (*t*(244) = 3.97, *p* < .001, *b* = .34, *SE* = .09).

These results indicate that the more the participants in our study experienced the intervention messages to be humorous in combination with higher levels of perceived cognitive fatigue, the more the intervention message positively influenced their intentions for brisk walking, accepting our second hypothesis.

#### Potential mediators self-efficacy, Self-Control & Motivation (H3_a_, H4_a_ and H5_a_)

**Fig. 5:**
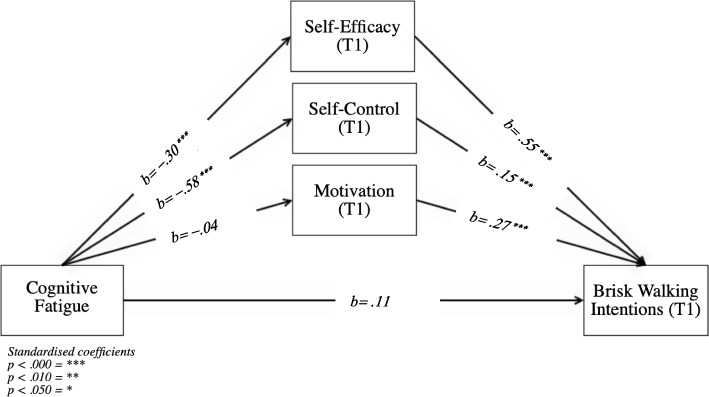
Visual presentation of H3_a_, H4_a_, H5_a_

The overall mediation model (Fig. 5) was statistically significant (R^2^ = 0.60, *F*(4, 244) = 93.14, *p* < 0.001). Based on the standardised indirect effects of cognitive fatigue on brisk walking intentions (T1), we can conclude that self-efficacy (T1) (*b* = −.16, *SE* = .04; BC 95% *CI* [−.24 to −.09]) and self-control (T1) (*b* = −.09, *SE* = .03; BC 95% *CI* [−.16 to −.03]) are significant mediators, however, motivation (T1) is not (*b* = −.01, *SE* = .0; BC 95% *CI* [−.05 to .03]). The direct effects of cognitive fatigue on self-efficacy (*t*(247) = − 4.86, *p* < .001, *b* = −.30, *SE* = .04]) and self-control (*t*(247) = − 11.30, *p* < .001, *b* = −.58, *SE* = .04]) are significant and negative, meaning that the more an individual feels cognitively fatigued, the less self-efficacy and self-control they will experience. Higher levels of self-efficacy (*t*(244) = 11.11, *p* < .001, *b* = .55, *SE* = .09]) and self-control (*t*(244) = 2.80, *p* = .005, *b* = .15, *SE* = .09]) are directly and positively related to intentions to go for a brisk walk (T1), as is motivation (*t*(244) = 6.01, *p* < .001, *b* = .27, *SE* = .11]).

The direct effect of cognitive fatigue on walking intentions (T1) is insignificant (*t*(247) = 1.75, *p* = .081, *b* = .11, *SE* = .06]). Cognitive fatigue does not significantly affect motivation (*t*(247) = −.62, *p* = .535, *b* = −.02, *SE* = .03]). Based on these results we can accept hypotheses H3_a_ and H4_a_, however rejecting H5_a_.

#### Moderation of perceived humour of the intervention messages that promote brisk walking behaviours (H3_b_, H4_b_ and H5_b_)

**Fig. 6:**
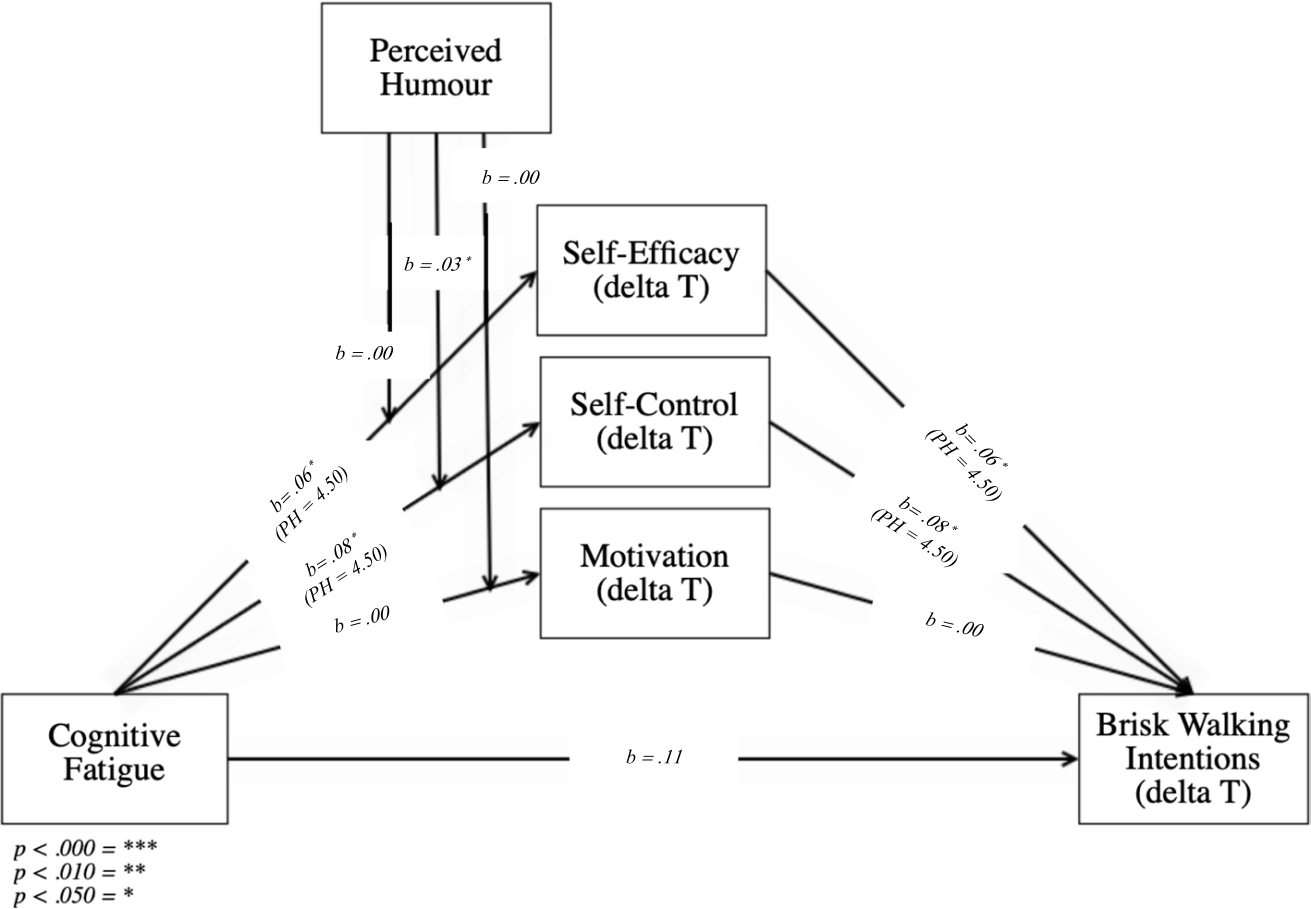
Visual presentation of H3_b_, H4_b_, H5_b_

When looking at the index of moderated mediation, the model (Fig. 6) shows a significant interaction for the α-path and a significant β-path for self-control (ΔT) (*b* = .01, *SE* = .01; BC 95% *CI* [.00 to .04]), but not for self-efficacy (ΔT) (*b* = .00, *SE* = .01; BC 95% *CI* [−.02 to .02]) and motivation (ΔT) (*b* = .00, *SE* = .00; BC 95% *CI* [−.01 to .01]) indicating a significant moderated mediation for self-control (ΔT), but not for self-efficacy (ΔT) nor motivation (ΔT).

The model indicates that the interaction between cognitive fatigue and perceived humour on self-control (ΔT) is positive and significant (*t*(245) = 1.98, *p* = .050, *b* = .03, *SE* = .01), more specifically, according to the Johnson-Neyman significance regions, only when perceived humour scores at least 2.01 out of seven points, perceived humour and cognitive fatigue are significantly related (*t*(245) = 1.97, *p* = .050, *b* = .08, *SE* = .04), meaning that the interaction only takes place when individuals perceive the messages humorous enough (i.e., 2.01 out of seven points). As perceived humour increases, the relationship between cognitive fatigue and perceived humour becomes more positive, with seven being the highest possible value for perceived humour (*t*(245) = 4.71, *p* < .001, *b* = .22, *SE* = .05). This indicates that the more individuals feel cognitively fatigued in combination with the more they experience the messages to be humorous, the more they perceive a positive change in levels of self-control (R^2^ = 0.01, *F*(1, 245) = 3.90, *p* = 0.050).

As a result, we can conclude that there is a moderated mediation of perceived humour on the relationship between cognitive fatigue and self-control (ΔT), but not on the relationship between cognitive fatigue and self-efficacy (ΔT) or cognitive fatigue and motivation (ΔT). These results indicate that hypothesis H4_b_ is supported, however hypotheses H3_b_ and H5_b_ are not. Further, the model indicates a significant mediation for self-efficacy (ΔT) (Perceived humour = 4.50, *b* = .06, *SE* = .03; BC 95% *CI* [.01 to .12]) and self-control (ΔT) (Perceived humour = 4.50, *b* = .08, *SE* = .03; BC 95% *CI* [.02 to .14]) starting from a value of 4.50 for perceived humour, but not for motivation (ΔT), which is insignificant at all levels (Perceived humour = 1.75, *b* = .00, *SE* = .01; BC 95% *CI* [−.02 to .04]). The direct effect between cognitive fatigue and brisk walking intentions (ΔT) is insignificant (*t*(244) = 1.68 *p* = .095, *b* = .11, *SE* = .07).

## Discussion

The current study had four objectives: First, we wanted to examine if feelings of cognitive fatigue negatively predicted brisk walking intentions (H1). Secondly, this paper tried to unravel the process through which this happened. Therefore, we investigated if self-efficacy, self-control, and motivation mediated the relationship between cognitive fatigue and intentions to brisk walk (H3_a_, H4_a_, H5_a_). Thirdly, we wanted to understand if intervention messages that are perceived as humorous moderate the relationship between feelings of cognitive fatigue and brisk walking intentions (H2). Lastly, we also wanted to examine if these (potential) mediation effects were influenced by viewing intervention messages that were perceived as humorous, thus examining moderated mediation of perceived humour of the intervention messages that promote brisk walking behaviours on the aforementioned mediations of self-efficacy, self-control and/or motivation (H3_b_, H4_b_, H5_b_).

This study confirms that cognitive fatigue negatively predicts physical activity intentions, more specifically brisk walking intentions (H1), which was also reported by several studies in the past [12, 19, 37]. Next, our results showed that the perceived humour of the intervention messages that promote brisk walking behaviours moderates the relationship between cognitive fatigue and brisk walking intentions, so that humorous messages become more effective in promoting physical activity, compared to non-humorous messages as cognitive fatigue increases (H2). Thus, we can conclude that humour appears to be beneficial in encouraging exercise in those who feel cognitively fatigued opening possibilities to tackle the problem of worldwide inactivity. However, to develop a full picture of how humorous messages are effective in motivating cognitively fatigued individuals to brisk walk, additional studies will be needed to expand the age category, ethnic grouping, and education levels. This is because the perception of humour is dependent on multiple demographic factors [52]. When we can understand what types of humour work best for what groups, we will be able to better individualise smartphone-based interventions in the future. Further, our study examined mediation effects for self-efficacy (H3_a_), self-control (H4_a_) and motivation (H5_a_) and found that self-efficacy (H3_a_) and self-control (H4_a_) mediated the relationship between cognitive fatigue and brisk walking intentions. However, no significant effects were observed for motivation (H5_a_). The mediation effect of self-efficacy fulfils expectations as it was already previously found within patient populations [60] and suggested to be researched within healthier populations [29, 36]. The mediation effect of self-control confirms the expected associations between cognitive fatigue, self-control, and brisk walking intentions based on previous findings [16, 22, 65]. These findings provide an opening into the investigation into other variables, next to self-efficacy and self-control, who might (even better?) explain why cognitively fatigued individuals are not willing to exercise. Furthermore, the mediated moderation model (H3_b_, H4_b_, H5_b_) showed only a significant mediated moderation for self-control (H4_b_), stating an interaction between cognitive fatigue and perceived humour of the intervention messages that promote brisk walking behaviours on self-control. As the study of Cheng and Wang [30] already found a causal impact of humour on persistence for cognitively fatigued individuals, and persistence is only possible when exerting sufficient self-control [66], these findings correspond to expectations based on previous findings. A future study with a focus on different physical activities, considering different intensity levels, is suggested to understand if these findings (the mediations and the moderated mediation of self-control) are related to the type of physical activity or not.

A possible explanation for the repeated significance of self-control in the tested models might be due to the high cohesion between the concepts of cognitive fatigue and self-control. As such, definitions of cognitive fatigue and self-control are sometimes rather closely aligned, centred around either the problem of control and/or the management of energetic resources. For instance, some scholars refer to cognitive fatigue as the result of a conflict between competing goals, more specifically a competition between doing one thing or another [92]. Similarly, self-control is mostly defined as “*the capacity of the individual to alter, modify, change, or override his or her impulses, desires, and habitual responses”* [93] – and according to this theory, “*self-controlled behaviour is voluntarily choosing for the personally valued longer-term goal despite conflicting urges that are more potent in the moment”* [64]. With regard to the management of energy, both cognitive fatigue and self-control are sometimes viewed as a loss of energy caused by the exhaustion of mental resources – both are depicted as batteries that can run down [94–96]. In conclusion, both cognitive fatigue and self-control appear to be depleted resources when having a hard time choosing between a long-term investment goal, such as taking a brisk walk, and a short-term ‘treat’, such as watching television [65, 97]. Although their similarities, this paper is not trying to claim both concepts are equal. As such, the correlations between both concepts were moderate and negative, indicating that when one variable increases, the other decreases. Moreover, studies clearly state a direct relationship between both concepts, such as Clarkson and colleagues [22] who report that individuals who are less cognitively fatigued elicit more self-control, which is similar to our findings. Nevertheless, more research is recommended concerning the differences and similarities between cognitive fatigue and self-control, and more specifically about their relationship to physical activity intentions and subsequent behaviours. Lastly, the parallels between cognitive fatigue and self-control may underlie why the perceived humour of the intervention messages that promote brisk walking behaviours have similar effects on cognitive fatigue and self-control. However, as self-efficacy was also found to be a significant mediator between cognitive fatigue and physical activity intentions, other types of messages might be effective in interacting with cognitive fatigue and at the same time increasing levels of self-efficacy. Further research can examine other types of humour or other content of messages overall.

### Limitations & strengths

The current study is subject to certain limitations. For instance, this study was an online experiment, which means the design did not permit the researchers to measure actual behaviours, but rather intentions. Further, within this study we have focused on one particular form of moderate-intensity physical activity, namely brisk walking. As such, results concerning other moderate-intensity activities may be different, possibly also when participants are allowed to choose their own activity. In addition, we used cat memes as stimulus materials for humorous messages within this experiment. As a result, we are unable to generalise about different types of humour. Although participants’ perceptions of humour were solicited, their real preferences for humor - or physical activity messages generally - were not taken into consideration.

Nevertheless, as the main aim of this paper was to examine underlying effects, the design chosen is the most appropriate to examine these processes and ensure high external validity at the same time. In addition, one of the greatest strengths of our study is the success of the manipulation and thereby being able to investigate brisk walking intentions under a particular state. Another strength of this study was the repeated measurement of the same variables (self-efficacy, self-control, and motivation) at different points in time (before and after the messages were viewed) allowing causal effects to be attributed to the messages.

### Practical implications

Interventions aiming at promoting physical activities like brisk walking can be considerably more effective when taking into account the impact of cognitive fatigue, self-efficacy, and self-control in brisk walking intentions. Health professionals, educators, and policymakers can use this knowledge to design workshops and campaigns that inform participants about the negative effects of cognitive fatigue on physical activity while also offering strategies to combat it by enhancing self-efficacy and self-control when it comes to brisk walking. Health professionals can also create customised treatments for different groups using this knowledge. For instance, their advice can be tailored to a person’s level of self-efficacy, self-control, and cognitive fatigue. By understanding these mediating factors, interventions can be more precise and effective. As a result, our research adds to the ongoing demand for more precise targeting, customization, and personalization of physical activity interventions, particularly for physical activity messages within smartphone-based interventions [9, 98].

Further, this study demonstrates how humour can increase brisk walking intentions in people who are cognitively fatigued. This implies that using humour to motivate individuals to exercise can be a successful strategy for health and fitness programmes. Humour can be used in marketing campaigns and health and wellness programmes to make their message more appealing and compelling. Existing mobile applications, for instance, may employ humour as a motivator by adding humorous achievements and notifications for reaching milestones in brisk walking. Likewise, existing health trackers, such as Fitbit, can also incorporate humorous elements into its app and devices to make the user experience more engaging. For example, it could include funny achievement badges or humorous messages in order for users to reach their daily step goals. This can boost motivation and make the fitness journey more enjoyable. In other words, as opposed to exclusively relying on behaviour change techniques [99, 100], applications should focus more on specific barriers, such as cognitive fatigue, and integrate specific solutions within their messages, such as humour, which respond to those barriers.

Lastly, this study’s focus on the underlying process reveals that humorous messages can positively impact self-control, which, in turn, increases intentions to brisk walk. This insight can guide the development of interventions that specifically target self-control mechanisms. It suggests that interventions can be created to support people in strengthening their self-control abilities, making it simpler for them to stick to a regular brisk walking schedule. As a result, online and/or offline training modules can be created that explicitly target the improvement of self-control, for example, by monitoring a person’s self-control development and intention to brisk walk. Furthermore, data collected by tracking devices could be used to provide personalized recommendations based on a user’s self-control and cognitive fatigue levels. For instance, it could suggest different walking strategies for days when users report feeling fatigued, emphasizing the importance of self-control in maintaining consistent walking routines.

## Conclusions

This study is one of the first to explain why cognitive fatigue negatively influences brisk walking behaviours, as it shows that self-efficacy and self-control mediate the relationship between cognitive fatigue and brisk walking intentions. Further, the results of this study redisplay that humorous messages are effective in enhancing brisk walking intentions in cognitively fatigued individuals overall. Moreover, this study is the first to unravel this underlying process, and thereby the first to understand that humorous messages can create positive changes in levels of self-control, thereby increasing intentions to brisk walk.

### Supplementary Information

**Additional file 1.**

## Data Availability

The datasets generated and/or analysed during the current study are not publicly available due to participants only agreeing to share data with the contributing researchers, corresponding to the ethical guidelines applied by the University of Antwerp, but are available from the corresponding author on reasonable request.

## Footnotes

1 Brisk walking was defined as follows: *a moderate-intensity physical activity where one walks at an average pace of 3.5 miles/hour - or, on average, 100 steps per minute. It increases heart rate and breathing rates but does not render one* out of breath. A single brisk walk lasts 20–30 minutes.

2 Fatigue was explained as a combination of physical and cognitive fatigue, explaining this study was only interested in the cognitive part of fatigue, namely: *Cognitive fatigue can be better defined as mental weakness - if one has been doing something tedious like reading or calculating numbers all day long, they would most likely feel mentally drained afterwards*.
