# Supplementary material for: Boosts for walking: how humorous messages increase brisk walking among cognitively fatigued individuals
Source: BMC Public Health. 2024 Jan 9;24:128. doi: 10.1186/s12889-023-17464-z (PMC10775479; doi:10.1186/s12889-023-17464-z)
Supplement: Supplementary file 1 — Additional file 1. [file 12889_2023_17464_MOESM1_ESM.pdf]

Supplementary Materials

Stimuli Materials: Humorous and Non-Humorous Intervention Messages

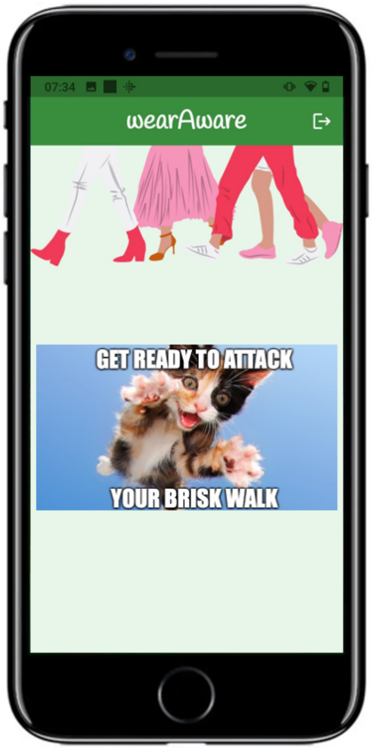

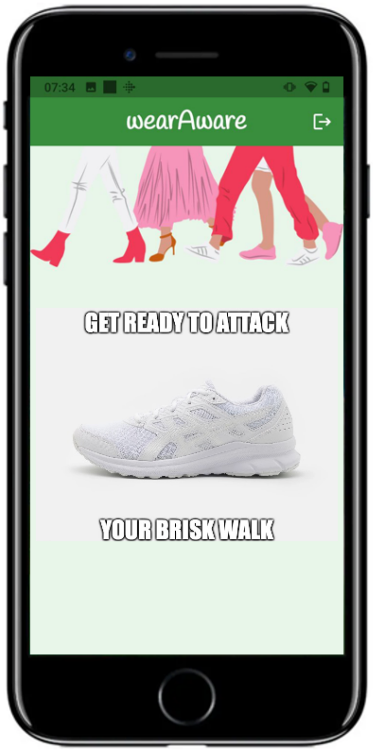

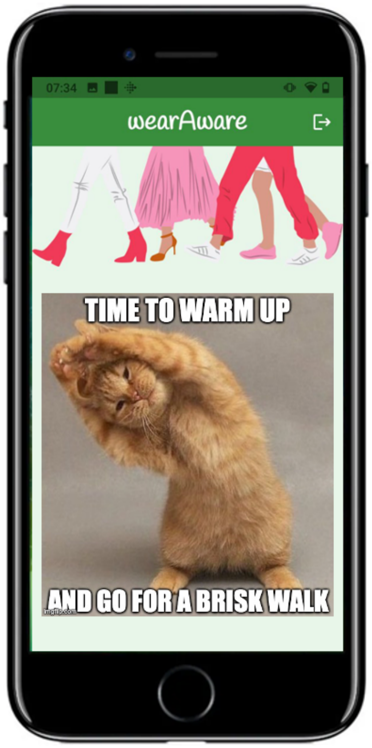

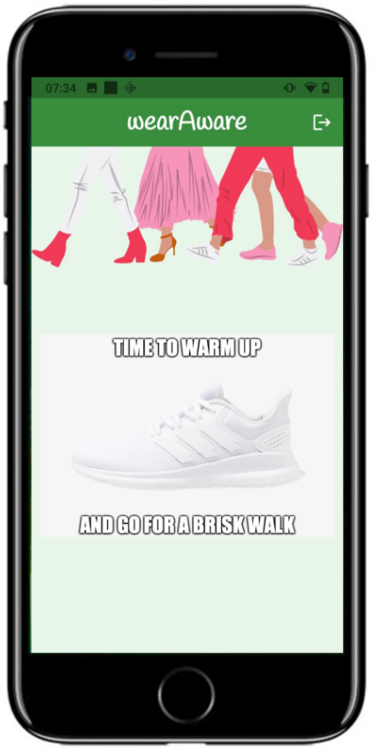

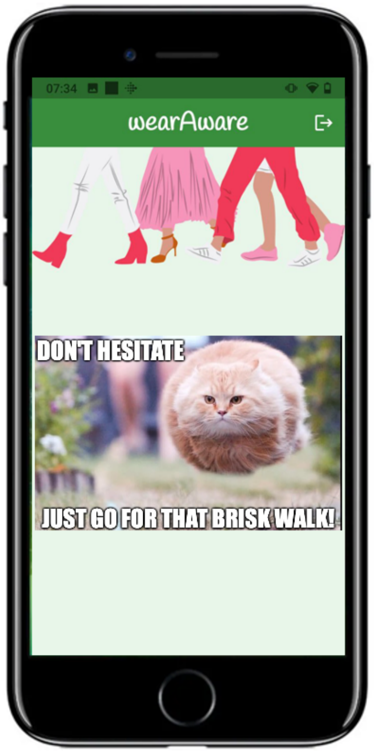
07:34
wearAware
DON'T HESITATE
JUST GO FOR THAT BRISK WALK!

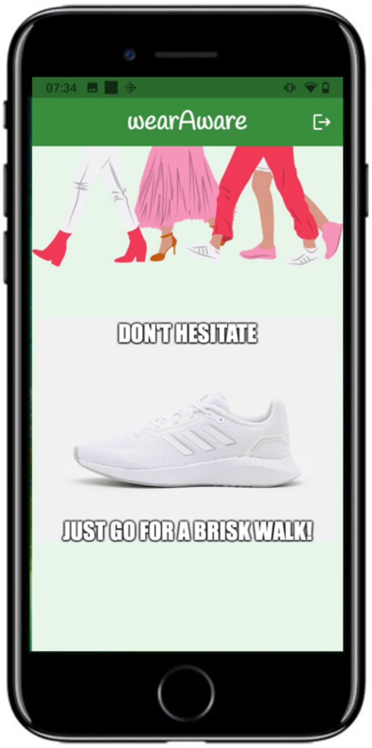
07:34
wearAware
DON'T HESITATE
JUST GO FOR A BRISK WALK!

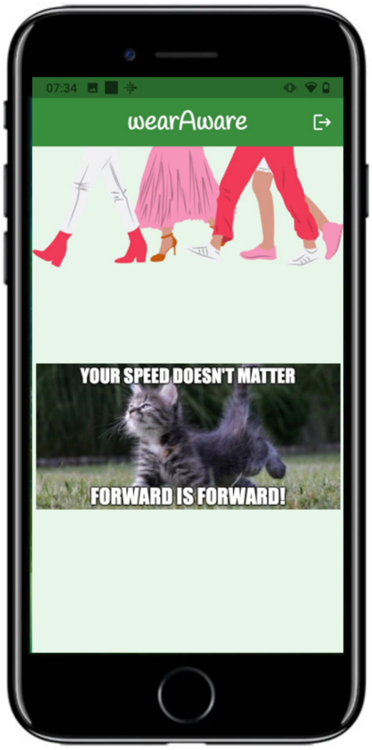
07:34
wearAware
YOUR SPEED DOESN'T MATTER
FORWARD IS FORWARD!

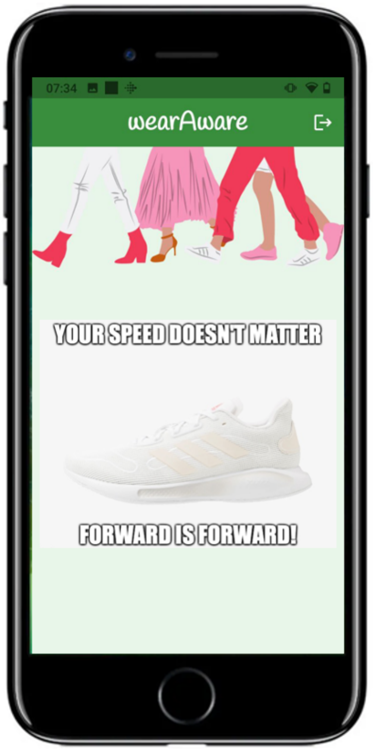
07:34
wearAware
YOUR SPEED DOESN'T MATTER
FORWARD IS FORWARD!

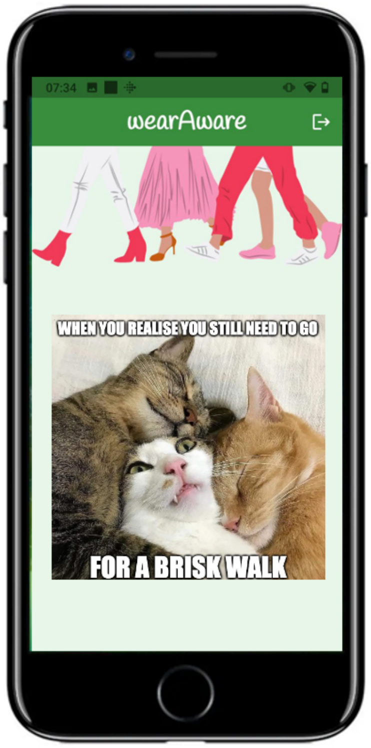
07:34
wearAware
WHEN YOU REALISE YOU STILL NEED TO GO
FOR A BRISK WALK

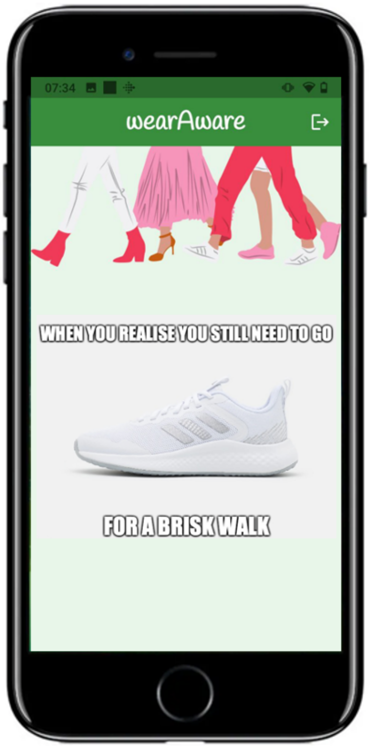
07:34
wearAware
WHEN YOU REALISE YOU STILL NEED TO GO
FOR A BRISK WALK

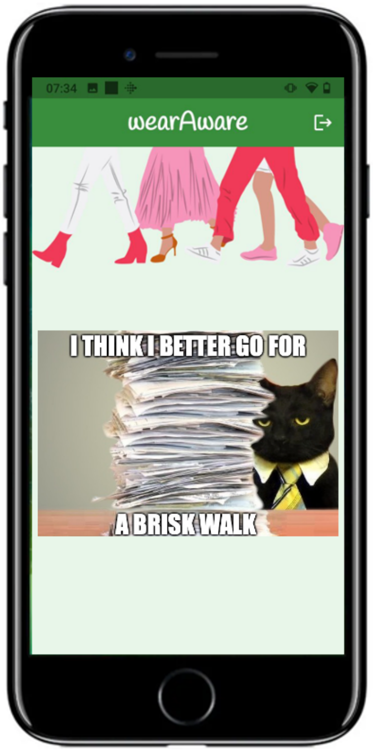
07:34
wearAware
I THINK I BETTER GO FOR
A BRISK WALK

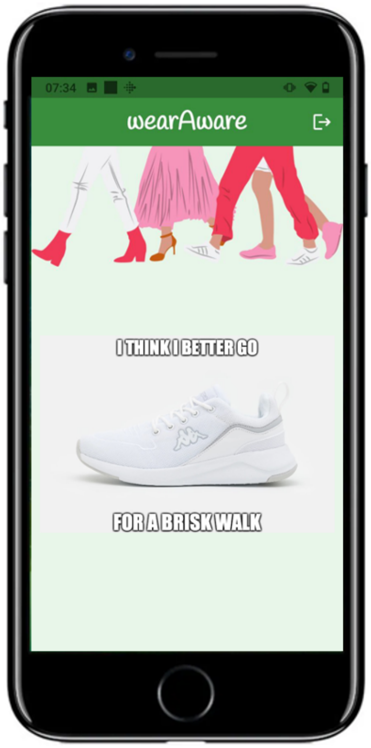
07:34
wearAware
I THINK I BETTER GO
FOR A BRISK WALK

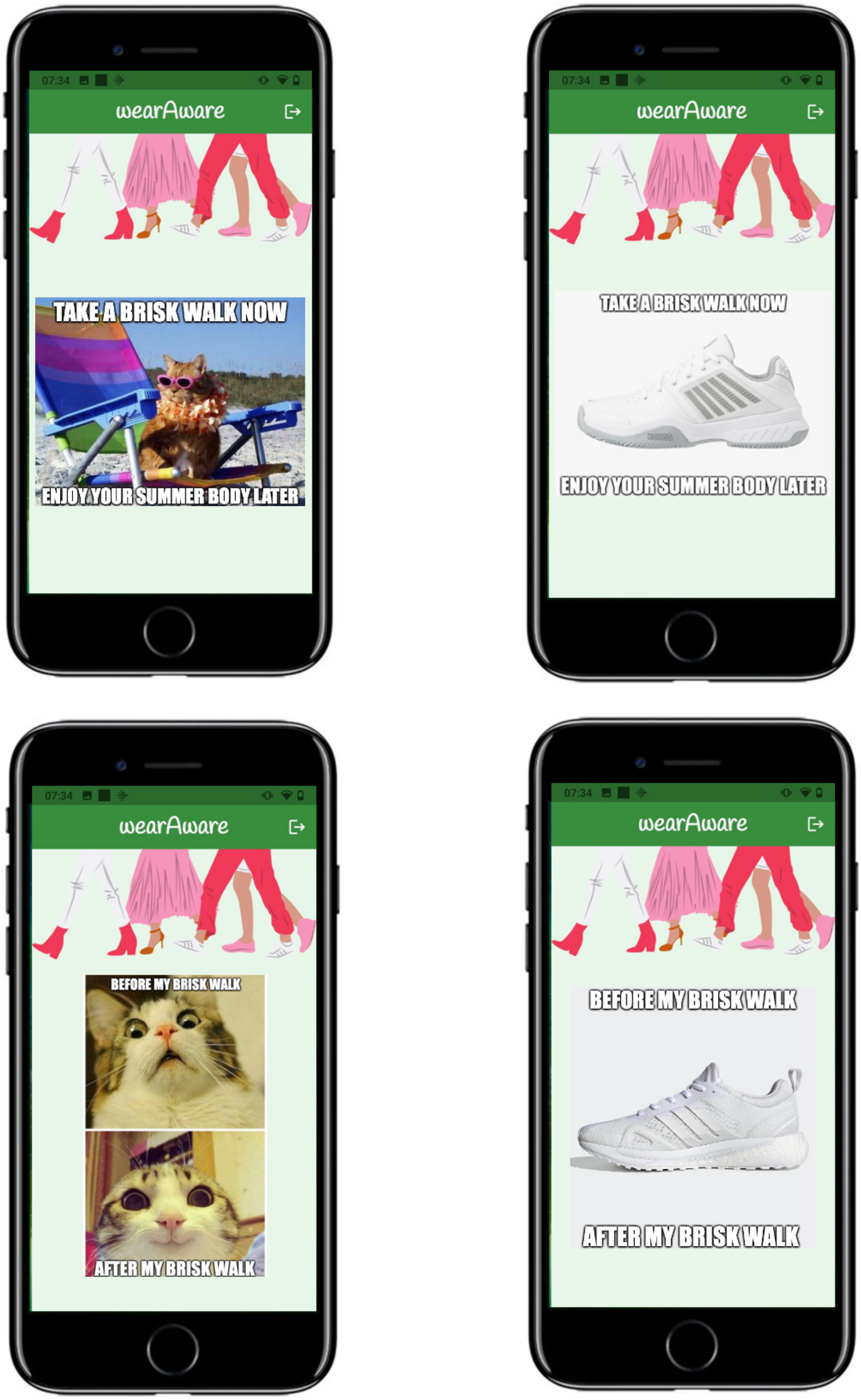
07:34
wearAware
TAKE A BRISK WALK NOW
ENJOY YOUR SUMMER BODY LATER
07:34
wearAware
TAKE A BRISK WALK NOW
ENJOY YOUR SUMMER BODY LATER
07:34
wearAware
BEFORE MY BRISK WALK
AFTER MY BRISK WALK
07:34
wearAware
BEFORE MY BRISK WALK
AFTER MY BRISK WALK

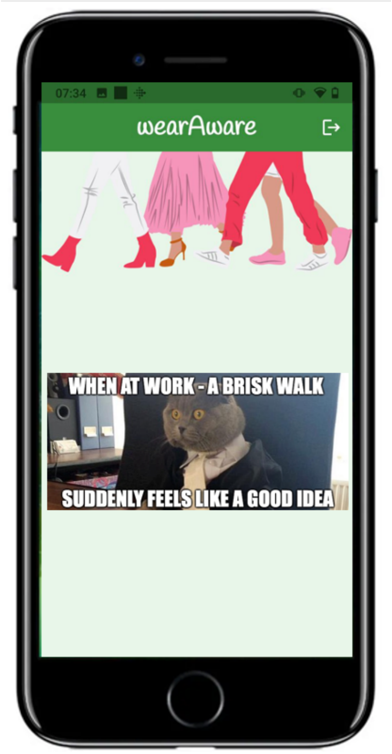
07:34
wearAware
WHEN AT WORK - A BRISK WALK
SUDDENLY FEELS LIKE A GOOD IDEA

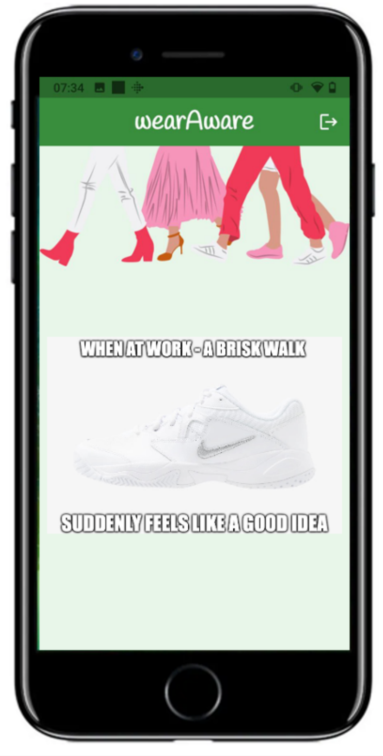
07:34
wearAware
WHEN AT WORK - A BRISK WALK
SUDDENLY FEELS LIKE A GOOD IDEA
